# Use of Biochar Prepared from the Açaí Seed as Adsorbent for the Uptake of Catechol from Synthetic Effluents

**DOI:** 10.3390/molecules27217570

**Published:** 2022-11-04

**Authors:** Uendel dos Santos Feitoza, Pascal S. Thue, Eder C. Lima, Glaydson S. dos Reis, Navid Rabiee, Wagner S. de Alencar, Beatris L. Mello, Younes Dehmani, Jörg Rinklebe, Silvio L. P. Dias

**Affiliations:** 1 Institute of Exact Sciences, Federal University of the South and Southeast of Pará (UNIFESPA), Marabá 68570-590, PA, Brazil; 2 Institute of Chemistry, Federal University of Rio Grande do Sul (UFRGS), Porto Alegre 91501-970, RS, Brazil; 3 Department of Forest Biomaterials and Technology, Swedish University of Agricultural Sciences, 245, 901 83 Umeå, Sweden; 4 School of Engineering, Macquarie University, Sydney, NSW 2109, Australia; 5 Laboratory of Chemistry and Biology Applied to the Environment, Faculty of Sciences of Meknes, Moulay Ismail University, Meknes 50070, Morocco; 6 School of Architecture and Civil Engineering, Institute of Foundation Engineering, University of Wuppertal, Water- and Waste-Management, Soil- and Groundwater-Management, Pauluskirchstraße 7, 42285 Wuppertal, Germany

**Keywords:** açaí kernel, biochar, adsorption, a phenolic compound

## Abstract

This work proposes a facile methodology for producing porous biochar material (ABC) from açaí kernel residue, produced by chemical impregnation with ZnCl_2_ (1:1) and pyrolysis at 650.0 °C. The characterization was achieved using several techniques, and the biochar material was employed as an adsorbent to remove catechol. The results show that ABC carbon has hydrophilic properties. The specific surface area and total pore volume are 1315 m^2^·g^−1^ and 0.7038 cm^3^·g^−1^, respectively. FTIR revealed the presence of oxygenated groups, which can influence catechol adsorption. The TGA/DTG indicated that the sample is thermally stable even at 580 °C. Adsorption studies showed that equilibrium was achieved in <50 min and the Avrami kinetic model best fits the experimental data, while Freundlich was observed to be the best-fitted isotherm model. Catechol adsorption on ABC biochar is governed by van der Waals forces and microporous and mesoporous filling mechanisms. The *Q_max_* is 339.5 mg·g^−1^ (40 °C) with 98.36% removal of simulated effluent, showing that açaí kernel is excellent biomass to prepare good biochar that can be efficiently used to treat real industrial effluents.

## 1. Introduction

Water is an essential natural resource, a component of living beings, and essential to the life of various plant and animal species. However, water availability is increasingly threatened due mainly to industrial pollutants [1]. In addition, some factors have enormously contributed to water scarcity, such as increasing population, the growth of the agricultural and industrial sectors, and the disorderly discharge of industrial and urban effluents [2,3].

Organic pollutants, in particular, have caused alarming concerns in recent decades [4,5]. As a result, phenolic compounds are generally discarded as industrial waste from the wood, paint, pharmaceutical, and petroleum industries [6,7].

Phenols are water-soluble and toxic to the environment. Therefore, effluents containing phenolic compounds present severe environmental problems due to their high toxicity [6]. Furthermore, the intake of phenols in contaminated water can provoke muscle tremors, gastrointestinal damage, and death in living beings [8,9].

Due to their toxicity, phenol compounds are included in the Environmental Protection Agency’s (EPA) list of risk pollutants, as they have carcinogens and cause numerous toxic and chronic effects, such as headaches, vomiting, anorexia, and other disorders [8,9].

Various processes have been used to remove phenols from wastewater, including membrane separation [10], electrochemical oxidation [11], photocatalytic Fenton processes [12], and biological processes [13]. However, these methods have limited applications and are complex and expensive. Conversely, adsorption is a suitable method for the treatment of organic compounds containing wastewater [14,15,16] because it is a simple, economical process, and it enables the use of the lignocellulosic materials in the pristine form [17] or as precursors for the preparation of adsorbents [18,19,20]. In addition, these adsorbents can be used to remove substances dissolved in water [19,20].

Residual Amazonian fruits biomasses such as açaí [21], cupuassu [22], tucumã [23], and Pará chestnut [24] have been used for the production of adsorbents in adsorption processes. Açaí is a native Amazonian fruit produced by a palm tree called *Euterpe oleácea*. This fruit is essential for feeding the Amazonian population and of fundamental economic importance for many families that make their living from extractivism and fishing [21,25].

An estimate points out that Brazil in 2018 produced 221,000 tons of açaí; the state of Pará alone was responsible for 66% of this production. However, about 85% to 95% of fruit waste is discarded after processing [26].

The pulp removed from the fruit’s epicarp and mesocarp is the main form of commercialization of açaí. However, the endocarp, commonly called the stone, is usually discharged after processing to remove the husk from the fruit. This waste is usually discharged, without any concern, into the environment, despite having several uses, such as making handicrafts, organic fertilizer, and animal feed [25]. Data from the Brazilian Institute of Geography and Statistics (IBGE) have indicated that the State of Pará, Brazil, in 2014 alone, produced 81,000 tons of açaí seeds [25].

In order to provide a better destination, as well as to add value to those residues and reduce the environmental impact, this work proposed using açaí seed biomass to prepare biochar material through the conventional pyrolysis process and its application as an adsorbent in the removal of the catechol from aqueous media. The produced biochar was characterized using several techniques: nitrogen adsorption/desorption isotherms, FTIR, TGA/DTG, CHN/O elemental analysis, Hydrophobicity Index (HI), and the zero point of charges (pH_pzc_). Furthermore, the batch contact adsorption process was used to perform the adsorption studies. In addition, studies of batch contact adsorption (kinetic and equilibrium at different temperatures) were also performed to find the uptake mechanism between catechol and biochar carbon.

## 2. Results and Discussion

### 2.1. Textural Characteristics

**Figure 1:**
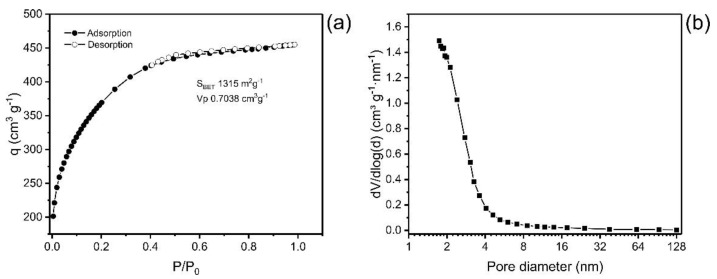
(**a**) Isotherm of adsorption and desorption of N_2_; (**b**) Pore size distribution curve.

Pore size distribution (PSD) and the specific surface area (S_BET_) are two fundamental parameters that affect the quality and usefulness of material for the adsorption process [27,28,29]. In addition, several studies have addressed the efficiency of an adsorbent as closely linked to specific surface area and pore size distribution [15,23]. Figure 1 presents the plot of the isotherm of adsorption/desorption of nitrogen and the distribution curve of the size of the pores. The values of textural characteristics of ABC carbon material are depicted in Table 1.

The values obtained for the textural surface of the ABC material were S_BET_ 1315 m^2^·g^−1^, the area assigned to micropores was 170 m^2^·g^−1^_,_ and the external surface area was 1145 m^2^·g^−1^. The format of the isotherm of adsorption of N_2_ is compatible with Type IA, which is characteristic of predominantly microporous materials [30].

The curve of the pore size distribution of the carbon material presents a maximum at pores < 2 nm, and it decreases exponentially as the pore size increases, showing that the material is predominantly microporous pores (ϕ < 2 nm), with a small portion of mesopores (2 < ϕ < 50 nm) and a tiny proportion of macropores (ϕ > 50 nm) (Figure 1b) [30].

ABC had high surface area (S_BET_) and total pore volume (V_total_) values compared with other materials presented in the literature. For example, Tran et al. [31] produced activated biochar utilizing pomelo peel as a carbon precursor, and the biochar presented a surface area ranging from 1033 to 1292 m² g^−1^; Hu et al. [32] produced sludge-derived biochar that was chemically activated with NaOH and KOH, obtaining S_BET_ of 756–814 m^2^ g^−1^; Quin et al. [33] prepared activated biochar from rape straw that was activated with KOH in different proportions of activating agent, obtaining surface areas from 1002 to 1531 m^2^ g^−1^ and total pore volume of 0.64 to 1.03 cm^3^ g^−1^. Therefore, the values of the textural characteristics of ABC carbon material agree with previous results of activated biochars.

This result also indicates that employing the ZnCl_2_ as activating agent can enhance the surface area and the pore volume of the biochar material, characterized by an increase in the nitrogen adsorption amount [15].

The SEM images of ABC material are presented in Figure S1. Irregular pieces of carbon material with different formats were obtained. The SEM images show that ABC presents some rugosity and some channels that allow solvent passage. Clearly, it is seen that the ABC material presents particles higher than 25 µm (estimated by SEM images), demonstrating that the carbon material could not be classified as a nanomaterial.

### 2.2. TGA Analysis

**Figure 2:**
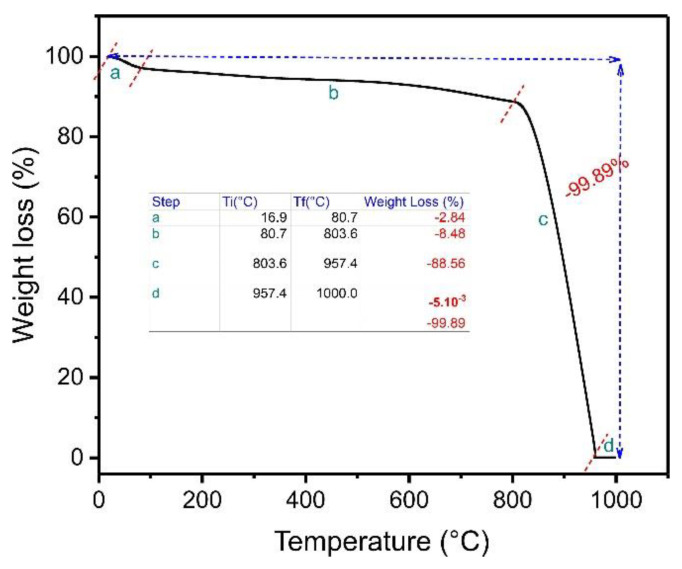
Thermogravimetric analysis of ABC biochar.

TGA is carried out to check samples’ thermal degradation or stability in a defined atmosphere. In this case, TGA was performed on an ABC sample. The experiment was conducted in a N_2_ atmosphere from ambient temperature up to 800 °C and using a synthetic air atmosphere from 800 °C to 1000 °C. At 1000 °C, carbon’s complete decomposition occurs, resulting in inorganic residues that can be attributed to ashes [15]. Figure 2 presents the thermal degradation of the biochar material. The curve presents four stages.

The first stage, ranging from 16.9 °C to 80.7 °C, corresponds to the weight loss of the absorbed water in the carbon matrix ABC [23]. The weight-loss percentage in this first step was about 2.84%. The second weight-loss step ranged from 80.7 to 803.6 °C, where the weight-loss percentage was 8.48%. This result demonstrates that the material is thermally stable in this temperature range, favoring its use even at high temperatures [23]. The third stage of weight loss (803.6–957.4 °C) corresponds to the decomposition of the ABC matrix in the presence of oxygen, presenting a major weight loss of 88.56%. A very slight weight loss was observed from 957.4 to 1000 °C (−0.005%). The total weight loss was 99.89%, meaning only a small residual mass was left (0.11%), corresponding to the ash content since the air atmosphere was used from 800 °C to 1000 °C [23].

### 2.3. Infrared Spectroscopy (FTIR)

**Figure 3:**
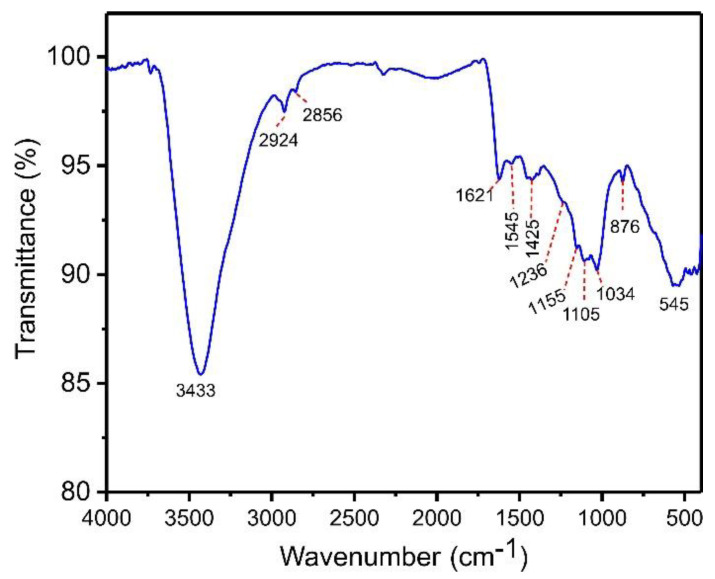
FTIR spectra of ABC biochar.

Chemical characterization of functional groups in the bulk phase and on the surface of the biochar material was studied using a Fourier Transform Infrared (FTIR) spectrometer (Bruker, model alpha) in the range 4000–400 cm^−1^. This analysis enables a better understanding of the material’s surface characteristics needed for more effective adsorption. The main components of açaí biomass are hemicellulose, cellulose, lignin, and tannin. Thus, activated biochar may present functional groups on its surface, including phenols, carboxylic, esters, and alcohols, which are involved in the adsorption process [34]. Figure 3 shows the infrared spectrum of ABC biochar. The intense and wide band at 3433 cm^−1^ is ascribed to stretching N-H or O-H moieties [34]. The peaks found at 2856 and 2924 cm^−1^, respectively, are attributed to symmetric and asymmetric C-H groups’ stretches [34]. The band at 1621 cm^−1^ is assigned to the stretch of O=C carboxylate [34]. The tiny bands at 1545 and 1425 cm^−1^ are attributed to the ring mode of arene compounds [34]. The shoulder at 1236 cm^−1^ is attributed to the C-C-C stretching of aryl–alkyl groups [34]. The band at 1155 and 1105 cm^−1^ corresponds to the C-C-O of esther or C-O-C of ether stretching [34]. The band at 1034 cm^−1^ is ascribed to C-O of phenolic groups or carboxylate stretch [34]. Finally, the band at 876 cm^−1^ is attributed to the –CH out-of-plane bending of aromatics [34]. The band at 545 cm^−1^ can be assigned to Zn-O left in the carbonaceous matrix [34].

Based on this founding, it is possible to say that ABC material showed several functional groups at its surface that may be bound with catechol in aqueous media by hydrogen bonds or π—π interactions principally. Furthermore, the material could adsorb other molecules and pollutants [16,27].

### 2.4. Elemental Analysis

The results of the elemental analysis of ABC carbon are shown in Table 2.

Compared to the açaí biomass material, ABC exhibited a very high amount of carbon content and a small amount of oxygen content. In fact, açaí biomass initially exhibited 43.29% carbon, 5.98% hydrogen, 1.25% of nitrogen, and 47.59% oxygen, but after carbonization in an inert atmosphere and washing process, the carbon content increased to 72.20%, while oxygen content decreased to 22.73% (See Table 2). Indeed, higher carbon content indicates that the aromatic structure becomes dominant after carbonization in the presence of zinc chloride. It was also possible with TGA analysis to determine the ash containing the ABC sample (0.11%). The ash content is attributed to the inorganic compound left after pyrolysis in the synthetic air atmosphere [15,23]. We can notice in Table 2 that the ash content was very small for the biomass precursor (1.89%) and only 0.11% for the prepared ABC material. This indicates that all the inorganics were removed after leaching, leaving the material with more pores [15,23]. Moreover, the very small value of ash content on açaí biomass (1.89%) may show the good potential of that residue to be used as a precursor in the preparation of biochar material or biosorbents.

### 2.5. Hydrophobicity Test (HI)

The hydrophobicity–hydrophilicity (HI) ratio, which was calculated from the quantity of vapor of n-heptane (mg·g^−1^) adsorbed divided by the quantity of vapor of water (mg·g^−1^) adsorbed, is intended to indicate the predominant affinity of the material for polar and non-polar solvents [15,29]. The HI is defined as:

(1) HI=amount of n−heptane vapor (mg)adsorbent mass (g)amount of water vapor (mg)adsorbent mass (g)

This ratio resulted in a value of 0.8420, which indicates that ABC has a greater affinity for water, thus being classified as hydrophilic [15,29].

This property could originate from the total functional groups composed of the acidic and basic groups. In fact, the functional groups such as carboxyl (–COOH), hydroxyl (–OH), carbonyl (C=O), amides (O=C-NH-), and amine (-NH_2_, -NH), available in the ABC biochar material could be associated with the uptake of H_2_O_(g)_ by the hydrogen-bound formation. Furthermore, observing the molecular volume of water (19.51 Å^3^) and n-heptane (130.0 Å^3^) obtained by van der Waals calculation, it is possible to state that the adsorption of H_2_O_(g)_ could be more favorable because of the micropore structures of ABC biochar material (see Table 1). In contrast, C_7_H_16(g)_ adsorption molecules will be complicated. This result is very similar to Thue et al. [27], who obtained HI < 1 for activated carbons prepared from sawdust, using ZnCl_2_ as an activating agent in their work. It is also highlighted that biochar materials with a high amount of C-content and the least O-content, resulting in a high level of aromatization during the pyrolysis process, tend to present hydrophobic properties. This hydrophilic behavior of ABC carbon material is important for the material to be a potential adsorbent to remove emerging organic contaminants.

Based on the ABC material’s characterization results, its chemical structure is proposed in Figure S2.

### 2.6. Zero-Charge Point (pH_pcz_)

**Figure 4:**
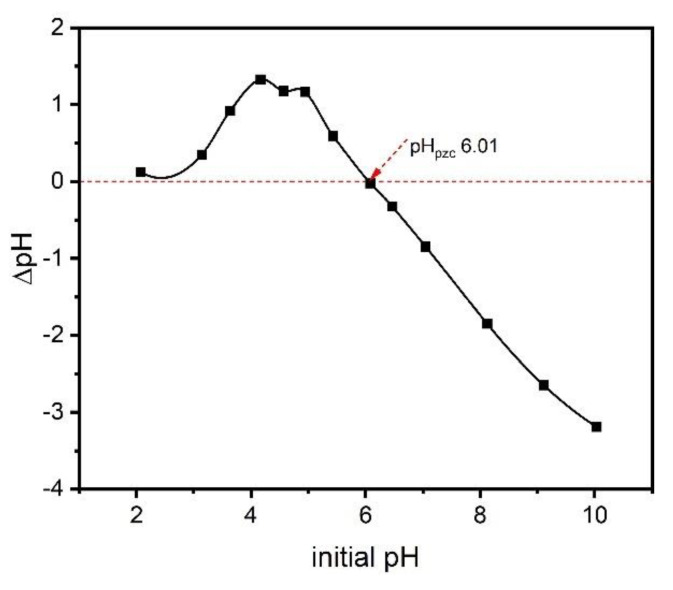
The pH_pzc_ of ABC.

The zero-charge point is an important parameter, as it shows the pH value at which a solid is zero electrically charged on its surface. The pH_pcz_ value of the adsorbent provides relevant information about the conditions under which the solid–liquid adsorption process takes place and how easily an adsorbent can absorb hazardous substances [27]. The surface of the adsorbent is negative when the pH of the solution exceeds the pH_pcz_ value and thus favors the adsorption of positively charged species, whereas when the pH of the solution is lower than pH_pcz_, the surface of the adsorbent will be positively charged and thus the adsorption of adsorbate with a negative charge is favored [27]. Figure 4 shows the pH_pzc_ of the ABC sample prepared with zinc chloride. The pH_pcz_ value of the biochar material was 6.01, which tends to be neutral. This result can be justified by the presence of both acid and basic groups at the material’s surface. The result also indicates that at pH 7, the pH of the preparation solution of catechol, the surface of the ABC biochar was negatively charged. Therefore, the adsorption process will be facilitated if the adsorbate molecules are positively charged in the solution.

### 2.7. Adsorption Kinetics

**Figure 5:**
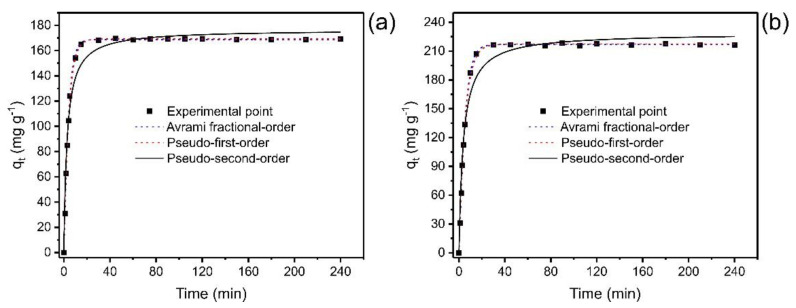
Kinetic curves for the adsorption of catechol. (**a**) Co 350 mg L^−1^; (**b**) 700 mg L^−1^. Adsorbent mass 30 mg; temperature 25 °C, pH 7.

To provide relevant information about the adsorption process, the study of kinetics is of fundamental importance [35,36,37,38]. The experimental operating conditions were pH 7.0, temperature 25 °C, under constant shaking (150 rpm). Figure 5 illustrates the relationship between the adsorption capacity at any time (q_t_) and the contact time. The data were fitted with nonlinear models that describe the kinetic behavior.

In the initial stages, the adsorption process is faster due to the many sites available; when the equilibrium approaches, adsorption becomes slower. Over time, the number of empty sites decreases, and the repulsive forces of the phenol molecules already adsorbed make the adsorption in the remaining sites difficult [37,38].

Figure 5 shows that the saturation time was below 40 min for both concentrations, which implies that this time is sufficient to reach the equilibrium for catechol adsorption. However, it is also possible to observe that concentration did affect the equilibrium time of the adsorption process when the catechol concentration was doubled (from 350 to 700 mg L^−1^). Therefore, nonlinear kinetic models of the pseudo-first-order, pseudo-second-order, and fractional-order of Avrami order [37] were applied to relate the experiment data with mathematical models available in the literature, thus predicting the adsorption behavior. The kinetic data were fitted to three kinetic adsorption models (Figure 5), and Table 3 shows the parameters of each model.

In order to validate the models and choose the one that best explains the adsorption phenomena, the adjusted determination coefficients (*R*^2^_*adj*_) and standard deviation of residuals (*SD*) were considered, where the lower *SD* values and higher *R*^2^*_adj_* tending to 1.0 suggest less disparity between the theoretical and experimental equilibrium adsorption capacities (q_t_) [37,38]. Among the three kinetic models employed to adjust the experimental kinetic data, the Avrami model showed the lowest standard deviation of the residues (*SD*) (ranging from 0.8897 to 1.402) and the highest *R*^2^_*adj*_ (ranging from 0.9993 to 0.9999) at both concentrations (see Table 3). This could confirm that q_t_ values obtained experimentally are closer to those found by the model. In addition, the BIC was also tested to check the statistical accuracy of the kinetic models [37,38]. When the ΔBIC of two models ≥10, the model with lower BIC values is certainly the best-fitted model [37,38]. The ΔBIC between PFO and Avrami and PSO and Avrami were 12.25–37.44 and 54.38–95.56, respectively. Based on these results, the Avrami fractional-order model best describes the kinetics of the uptake of catechol onto ABC carbon material.

The t_1/2_ and t_0.95_, considered the times needed to reach 50% and 95% of saturation, respectively [37], were obtained by interpolating into their respective kinetic curves shown in Figure 5 and Table 3. Considering that the Avrami fractional-order model was the best kinetic model to fit the experimental data, the most truthful values of t_1/2_ and t_0.95_ are based on this model. The values of t_1/2_ ranged from 2.922 to 3.790 min and t_0.95_ from 11.09 to 14.26 min (Avrami fractional model). Therefore, an increase in the initial concentration of adsorbate can increase the time needed to reach equilibrium [37]. Therefore, in further equilibrium studies, the contact time was fixed at 30 min.

### 2.8. Adsorption Isotherm

At constant temperature, adsorption isotherms are used to describe the relationship between the amount of adsorbate adsorbed by the ABC biochar (*q_e_*) and the remaining concentration of adsorbate in the solution after the system reaches equilibrium (C_e_) [37].

**Figure 6:**
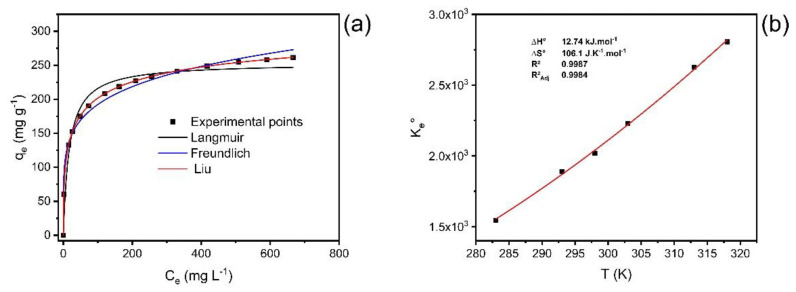
(**a**) Isotherm of adsorption of catechol at 40 °C; (**b**) nonlinear van’t Hoff plot for the calculation of the thermodynamic parameters.

The experiment data analysis was fitted using the Liu, Freundlich, and Langmuir equilibrium models, at temperatures between 10 °C and 45 °C under the following experimental conditions: 1.5 g·L^−1^ adsorbent dosage; initial pH of 7.0; and using a contact time of 30 min. The results of the isotherm curves and their respective adsorption parameters are shown in Figure 6 (temperatures from 40 °C) and Table 4.

The statistical analysis of the fitted models was based on *R*^2^*_adj_*, *SD*, and BIC values. The Liu isotherm model presented the values of *R²_adj_* closer to 1, the lowest values of *SD* and BIC [37,38]. As observed for the kinetic results, the ΔBIC values between the Langmuir and Liu and Freundlich and Liu models were always >10 (Table 4), suggesting that the uptake equilibrium of catechol onto ABC carbon material from 10 °C to 45 °C was better explained based on the Liu isotherm model.

The values of *Q_max_* for the uptake of catechol using ABC carbon material ranged from 243.6 to 339.5 mg·g^−1^, the maximum value obtained at 40 °C.

### 2.9. Adsorption Thermodynamics

Since temperature is an essential physicochemical variable that can affect adsorption, temperature variation directly impacts the efficiency of the adsorption of contaminants [39,40].

The thermodynamic study for catechol adsorption on ABC carbon material was performed at a temperature ranging from 10 °C to 45 °C (283 to 318 K) (Figure 6b; Table 5).

Based on the magnitude of enthalpy variation values, adsorption can be considered physical or chemical [37,39,40]. The process is considered chemical adsorption when the values of ∆H° are higher than 200 kJ·mol^−1^. The interactions can be classified as physisorption in the range of up to 80 kJ·mol^−1^ [37,39,40]. The ΔH° obtained was 12.74 kJ·mol^−1^, which is compatible with physisorption (van der Waals forces). Furthermore, the positive value of enthalpy changes demonstrates that the adsorption process is endothermic. The positive value of ∆S° indicates an increase in randomness at the solid–liquid interface during adsorption. This behavior is compatible with an increase in randomness due to the desolvation of adsorbate molecules before the carbon surface uptake. The ΔG° values were lower than zero for all studied temperatures. This behavior indicates that the adsorption process was favorable from 10 °C to 45 °C. Considering that the values of ΔH° were positive (endothermic), the catechol uptake onto ABC carbon material was controlled by the entropy changes (that were positive) [37,39,40].

### 2.10. Simulated Effluent Removal

Emerging contaminants, such as phenols, coming from industrial streams, are usually found amongst a complex matrix of effluents, a varied mixture of organic and inorganic compounds to salts and sugars.

**Figure 7:**
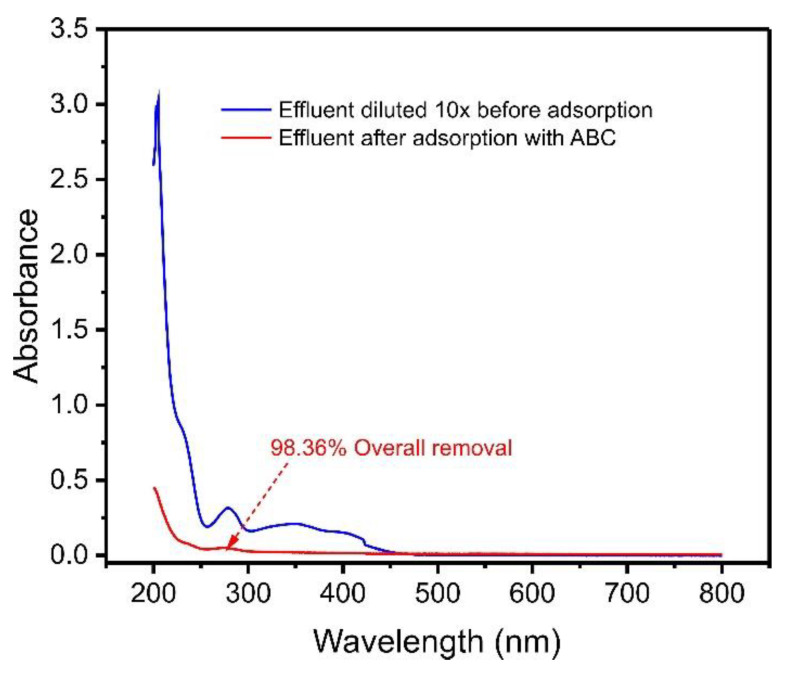
UV–VIS spectra of synthetic effluent before adsorption and after treatment with ABC biochar. For the composition of synthetic effluent, see Table S1.

Aiming to investigate the efficiency of ABC biochar in removing contaminants in an aqueous media, we simulated an effluent with varied compositions, as described in Table 1. As a result, the UV–VIS spectra of treated and untreated effluents were observed from 190 to 800 nm, as shown in Figure 7.

The removal percentage was calculated by the expression:

(2) $$Removal\;\left(\%\right)=100\cdot \frac{Integrated\;area\;after\;adsorption}{Integrated\;area\;before\;adsorption}\;$$

The integration of the absorption bands obtained from 190 to 800 nm of the synthetic effluents before and after the adsorption.

The spectral scan (Figure 7) indicated that after the adsorption process, the effluent decreased the intensity of its bands more than six times, corresponding to 98.36% adsorption of the contaminants in the solution. This result shows that açaí seed-based biochar material can be applied to remove compounds from real effluents contaminated with phenols.

Furthermore, the removal of catechol from the effluent was evaluated using a chemometric approach [41], and the removal of catechol in the simulated effluent attained 99.4% removal, indicating that ABC carbon material is efficient for the removal of catechol from simulated industrial effluents.

## 3. Materials and Methods

### 3.1. Biochar Carbon Preparation

Açaí seeds were washed with tap water and dried at 105 °C for 4 h. Afterward, the kernels were crushed in a knife mill in an analytical crusher and sieved using a 250 µm sieve.

Açaí-activated biochar (ABC) was prepared using 60 g of zinc chloride and 60 g of açaí seed mixed in a beaker with deionized water for 1 h until it became a homogeneous paste [27]. First, the temperature was set at 80 °C. Next, the paste formed was dried in an oven for 24 h at 80 °C. After drying, the paste was introduced into a quartz reactor in a vertical furnace, and it was heated step-by-step from 25 °C up to 650 °C utilizing a heating rate of 10 °C min^−1^ under a N_2_ atmosphere, with a flow rate of 200 mL min^−1^. When the furnace temperature reached 650 °C, the system was maintained for 30 min [14,15]. Subsequently, the heating system was shut down, while the N_2_ gas was maintained until the temperature became lower than 200 °C [14,15]. Afterward, 35 g of the carbonized material was mixed with 300 mL of a 6 mol L^−1^ HCl solution under reflux for 120 min at 90 °C to remove the zinc metal from the pyrolyzed material [14,15].

After leaching, the material was water-washed many times and filtered with a vacuum pump until obtaining a pH of about 6. Soon after, it was placed in an oven at 70 °C for 24 h. The obtained material was denominated as ABC.

### 3.2. Characterization of Açaí-Activated Biochar

ABC has been subjected to characterizations of textural characteristics and functional groups. These characterizations help to investigate the contributing factors of the physical–chemical characteristics of ABC material for being utilized as an adsorbent.

The obtained ABC material’s S_BET_ and V_total_ were quantified using N_2_ adsorption/desorption isotherms at −196.5 °C using an analyzer (Quantachrome NOVA 4200) [28].

Thermogravimetric (DTG) curves were obtained using a TA Instruments SDT Q 600 model. The material was analyzed using an inert atmosphere (N_2_) from ambient temperature up to 800 °C and an air atmosphere from 800 °C to 1000 °C using 10.00–15.00 mg of adsorbent [24].

The functional groups on the surface and bulk of the ABC biochar were found by FTIR spectroscopy using a Bruker alpha model instrument. The ABC sample was dried in an oven and stored in a desiccator until the analysis was performed, which was carried out with a spectral resolution of 4 cm^−1^ with 100 scans [27].

The carbon, hydrogen, and nitrogen elemental analysis were measured by employing a CHNS/O elemental analyzer (Agilent model 2400) [15,16].

The hydrophobicity index (HI) was determined as already reported [29]. In addition, the pH_pzc_ was obtained following the literature [14,15].

### 3.3. Adsorption Studies on ABC

Aqueous solutions of catechol (20 mL) with concentrations ranging from 200 to 1000 mg·L^−1^ were added to flat 50.0 mL conic polyethylene tubes containing 30 mg of ABC. Afterward, the tubes were capped and disposed of in a pendular shaker (Oxylab, São Leopoldo, RS, Brazil) with a thermostatic temperature, shaking at a speed of 120 rpm [14,15]; for further details, see the Supplementary Material.

The equilibrium experiment data were fitted with the nonlinear models of Freundlich and Langmuir, which are commonly employed in the literature [37,38]. The adsorption kinetics were fitted using the nonlinear pseudo-first-order, pseudo-second-order, and Avrami fractional-order models [37,38]; see the Supplementary Material.

The thermodynamic equilibrium constant values $K_{e}^{0}$ were acquired from the equilibrium studies from 283 to 318 K [39,40]. Values of the ΔH° and ΔS° of adsorption were calculated based on the nonlinear van’t Hoff equation [39,40]. See the Supplementary Material.

### 3.4. Synthetic Wastewater

A simulated effluent was prepared by mixing various inorganic and organic molecules detected typically in industrial waste [15,16] to evaluate the performance of the ABC biochar prepared from açaí kernels for potential application in wastewater treatment. The final pH was adjusted to pH 7.0. The composition and concentration of the different compounds used in the effluent preparation are shown in Table S1.

## 4. Conclusions

In this work, the seed of açaí fruit was used as a precursor material for producing biochar material in a conventional oven under an inert atmosphere, and ZnCl_2_ as the activating agent in the proportion of 1:1. The activated biochar obtained under these conditions had the following characteristics: total surface area of 1315 m^2^·g^−1^, total pore volume of 0.7040 cm^3^·g^−1^, and total mesopore volume of 0.6320 cm^3^·g^−1^. In addition, the elemental analysis showed that activated carbon has a high carbon content of 72.2%; the hydrophobicity test revealed the material to be predominantly hydrophilic; the zero charge point of ABC carbon is 6.01; FTIR showed that the carbon surface has oxygenated groups, which confers negative charge density on the surface that can establish interactions with phenol molecules, and thermogravimetric analysis revealed that the material has a certain thermal stability at a high temperature.

The kinetic study revealed that the catechol adsorption was fast initially, reaching 95% of saturation in less than 15 min for both concentrations at a temperature of 25 °C, the Avrami model being the one that best fit the experimental data.

Regarding the equilibrium study, it was noticed that at 40 °C, the maximum adsorption capacity was 339.5 mg·g^−1^, the maximum adsorption capacity of catechol based on the Liu isothermal model. The thermodynamic study showed that catechol uptake is a spontaneous, favorable, and endothermic process. The effluent test showed that ABC carbon has a sufficient adsorption capacity for industrial effluents containing phenols (removal >98%), and the individual removal of catechol from the simulated effluent attained 99.4% removal.

## Figures and Tables

**Table 1:** Specific surface area and pore volume of ABC carbon.

Parameters	Value
Total surface area (m^2^·g^−1^)	1315
Micropore surface area (m^2^·g^−1^)	170
External surface area (m^2^·g^−1^)	1145
Total pore volume (cm^3^·g^−1^)	0.7038
volume of micropores (cm^3^·g^−1^)	0.0720
volume of mesopores (cm^3^·g^−1^)	0.6318

**Table 2:** Elemental analysis of ABC carbon and raw açaí seed.

Sample	%C	%H	%N	% Ashes ^a^	%O ^b^
Raw açaí seed	43.29	5.98	1.25	1.89	47.59
ABC	72.20	3.33	1.63	0.11	22.73

^a^ Calculated from the TGA method. ^b^ Calculated by difference %O = 100% − (%C + %H + %N + %ashes).

**Table 3:** Kinetic parameters for adsorption of catechol onto ABC material. Conditions: m 30 mg; pH 7.0; temperature 25 °C.

*Co* (mg L^−1^)	350	700
**Avrami fractional-order**		
*q_e_* (mg·g^−1^)	168.7	216.8
*k_AV_* (min^−1^)	0.2450	0.1894
*n_AV_*	1.097	1.105
t_1/2_ (min)	2.922	3.790
t_0.95_ (min)	11.09	14.26
*R* ^2^_*adj*_	0.9993	0.9999
*SD* (mg·g^−1^)	1.402	0.8897
BIC	21.35	4.070
**Pseudo-first-order**		
*q_e_* (mg·g^−1^)	169.1	217.4
*k*_1_ (min^−1^)	0.2404	0.1842
t_1/2_ (min)	2.884	3.764
t_0.95_ (min)	12.46	16.27
*R* ^2^ *_adj_*	0.9986	0.9988
*SD* (mg·g^−1^)	2.028	2.498
BIC	33.60	41.51
**Pseudo-second-order**		
*q_e_* (mg·g^−1^)	176.6	228.8
*k*_2_ (g mg^−1^ min^−1^)	2.038 × 10^−3^	1.148 × 10^−3^
*t*_1/2_ (min)	2.716	3.692
t_0.95_ (min)	42.87	54.93
*R* ^2^ *_adj_*	0.9750	0.9752
*SD* (mg·g^−1^)	8.486	11.54
BIC	87.98	99.65

**Table 4:** Equilibrium parameters for the adsorption of catechol onto ABC material. Conditions: m 30 mg; pH 7.0; contact time of 30 min.

**Langmuir**	**10 °C**	**20 °C**	**25 °C**	**30 °C**	**40 °C**	**45 °C**
*Q_max_* (mg·g^−1^)	288.5	280.7	246.5	275.8	253.4	212.4
*K_L_* (L mg^−1^)	0.01203	0.01471	0.02421	0.02070	0.05768	0.1705
*R* ^2^ *_adj_*	0.9739	0.9637	0.9935	0.9999	0.9578	0.9064
*SD* (mg·g^−1^)	12.04	14.80	5.973	0.9009	15.75	19.74
BIC	80.62	86.83	59.59	2.848	88.69	95.46
**Freundlich**	**10 °C**	**20 °C**	**25 °C**	**30 °C**	**40 °C**	**45 °C**
*K_F_* (mg·g^−1^ (mg L^−1^)^−1/nF^)	42.63	45.47	47.07	49.36	82.23	102.1
*n* *_F_*	3.528	3.653	3.864	3.723	5.419	8.023
*R* ^2^ *_adj_*	0.8947	0.8696	0.9654	0.9442	0.9836	0.9923
*SD* (mg·g^−1^)	24.17	28.05	13.82	18.58	9.831	5.658
BIC	101.5	106.0	84.77	0.9442	74.54	57.97
**Liu**	**10 °C**	**20 °C**	**25 °C**	**30 °C**	**40 °C**	**45 °C**
*Q_max_* (mg·g^−1^)	249.3	243.6	274.3	279.0	339.5	334.2
*K_g_* (L mg^−1^)	0.01403	0.01715	0.01832	0.02024	0.02385	0.02548
*n* *_L_*	1.812	1.993	0.7533	0.9658	0.4385	0.2510
*R* ^2^ *_adj_*	0.9999	0.9999	0.9999	0.9999	0.9999	0.9999
*SD* (mg·g^−1^)	0.1331	0.1661	0.2429	0.4831	0.2712	0.5374
BIC	−53.01	−46.37	−34.97	−14.34	−31.66	−11.15

**Table 5:** Thermodynamic parameters for catechol adsorption at different temperatures.

Temperature (K)	283	293	298	303	313	318
$K_{e}^{0}$	1.545 × 10^3^	1.888 × 10^3^	2.018 × 10^3^	2.229 × 10^3^	2.626 × 10^3^	2.806 × 10^3^
∆G° (kJ·mol^−1^)	−17.28	−18.38	−18.85	−19.42	−20.49	−20.99
∆H° (kJ·mol^−1^)	-	-	12.74	-	-	-
∆S° (J·K^−1^ mol^−1^)	-	-	106.1	-	-	-

## Data Availability

Not applicable.

## Supplementary Materials

The following supporting information can be downloaded at: https://www.mdpi.com/article/10.3390/molecules27217570/s1, information about adsorption on ABC; Table S1: Chemical composition of simulated synthetic effluents; Figure S1: SEM images of ABC material, Figure S2: Chemical structure of ABC.

## Sample Availability

Samples of the compounds are not available from the authors.
