# Supplementary material for: Use of Biochar Prepared from the Açaí Seed as Adsorbent for the Uptake of Catechol from Synthetic Effluents
Source: Molecules. 2022 Nov 4;27(21):7570. doi: 10.3390/molecules27217570 (PMC9654046; doi:10.3390/molecules27217570)
Supplement: Supplementary file 1 [file molecules-27-07570-s001.zip › molecules-1991609-supplementary.pdf]

Supplementary Material

2.3-Adsorption Study on ABC

Aqueous solutions of catechol (20 mL) with concentrations ranging from 200 to 1000 mg L^{-1} were added to flat 50.0 mL Falcon tubes containing 30 mg of ABC. Afterward, the tubes were capped and arranged horizontally in an Oxylab shaker with a controlled temperature, shaking at a speed of 120 rpm (Leite et al., 2017; Carvalho et al., 2019; Thue et al., 2020).

The kinetic experiment was conducted using contact times from 1 to 240 min at 25 °C. For that purpose, two solutions of catechol were employed in concentrations of 350 $mg.L^{-1}$ and 700 $mg.L^{-1}$ and 20 mL of each solution were put in contact with 30.0 mg of adsorbent. Then, the samples were centrifuged in a UniCen M centrifuge (Herolab) for 5 min at 10,000 rpm to separate the ABC from the liquid phase. Finally, an aliquot of 1–10 mL of the supernatant was diluted adequately with an aqueous solution at pH 7.0.

The isotherm studies (200.0 and 1000.0 $mg.L^{-1}$) were carried out at temperatures ranging from 10 °C to 45 °C in a controlled temperature shaker for 24h. The final concentration of the remaining adsorbate was determined by visible spectrophotometry using a T90 + UV-VIS Spectrophotometer equipped with quartz cells. This equipment allows first to find the maximum wavelength of 266 nm corresponding to the catechol and measure the absorbance of the remaining absorbate in the solution (Leite et al., 2017).

The quantity of adsorbed catechol and the percentage of adsorbate removal by ABC is given by Equations S1 and S2, respectively:

$$q = \frac{(C_0 - C_f)}{m}.V \quad \text{(S1)}$$

$$\%\,Removal = 100.\frac{(C_0 - C_f)}{C_0} \quad \text{(S2)}$$

Where q is the adsorbed amount (mg/g); C_o is the initial concentration of catechol (mg/L); C_f is the final concentration of catechol (mg/L); V is the volume of adsorbate solution placed in contact with the adsorbent (L) and m is the mass of the adsorbent (g) (Carvalho et al. 2019; Thue et al. 2020)

The kinetic and equilibrium data's fitness was done using nonlinear methods, which were evaluated using the Simplex method and the Levenberg–Marquardt algorithm using the fitting facilities of the Microcal Origin 2021 software (Lima, E.C. et al., 2021a, 2021b). The suitableness of the kinetic and equilibrium models was evaluated using the residual sum of squares (*RSS*), the determination coefficient (R^2), the adjusted determination coefficient (R^2_{adj}), the standard deviation of residues (*SD*), and also the Bayesian Information Criterion (*BIC*) (Lima, E.C. et al., 2021a, 2021b). Equations S3 to S7 are the mathematical expressions for respective *RSS*, R^2, R^2_{adj}, *SD, and BIC*.

$$RSS = \sum_{i}^{n} \left(q_{i,exp} - q_{i,model}\right)^2 \qquad \text{(S3)}$$

$$R^2 = \left(\frac{\sum_i^n \left(q_{i,exp} - \bar{q}_{i,exp}\right)^2 - \sum_i^n \left(q_{i,exp} - q_{i,model}\right)^2}{\sum_i^n \left(q_{i,exp} - \bar{q}_{i,exp}\right)^2} \right) \qquad \text{(S4)}$$

$$R^2_{adj} = 1 - (1 - R^2).\left(\frac{n-1}{n-p-1}\right) \qquad \text{(S5)}$$

$$SD = \sqrt{\left(\frac{1}{n-p}\right).\sum_{i}^{n}\left(q_{i,exp} - q_{i,model}\right)^2} \qquad \text{(S6)}$$

$$BIC = nLn\left(\frac{RSS}{n}\right) + pLn(n) \qquad \text{(S7)}$$

In the above equations, $q_{i,\ model}$ is the individual theoretical *q* value predicted by the model; $q_{i,\ exp}$ is individual experimental *q* value; $\bar{q}_{exp}$ is the average of all experimental *q* values measured; *n* is the number of experiments; *p* is the number of parameters in the fitting model.

The values of R^2adj, SD, and BIC will be presented to compare different models of kinetics and equilibrium presented in this work. The best-fitted model would present R^2_{adj} closer to 1.000, lower values of SD, and BIC values. However, the kinetic and equilibrium model could not merely be chosen based on the values of R^2 (Lima, E.C. et al., 2021a, 2021b) when these models present a different number of parameters. Therefore, it is necessary to check if the R^2 values' improvements are due to the increase of a number of the parameters (Lima, E.C. et al., 2021a, 2021b) or if, physically, the model with more parameters explains better the process taking place (Lima, E.C. et al., 2021a, 2021b).

However, the difference in BIC values between models could be conclusive if the difference in BIC values ≤ 2.0, there is no significant difference between the two models (Lima, E.C. et al., 2021a, 2021b). When BIC values' difference is within 2–6, there is a positive perspective that the model with lower BIC is the most suitable (Lima, E.C. et al., 2021a, 2021b). For variations of BIC values from 6–10, there is a strong possibility that the model with a lower BIC value is the best model to be fitted (Lima, E.C. et al., 2021a, 2021b). However, if the difference in BIC values ≥ 10.0, it can be predicted with accuracy that the model with a lower BIC value is better fitted (Lima, E.C. et al., 2021a, 2021b).

Pseudo-first-order, pseudo-second-order, and Avrami fractional-order models (Lima, E.C. et al., 2021a) were used to fit the kinetic data. The mathematical equations of these respective models are shown in Equations S8, S9, and S10.

$$q_t = q_e.[1 - exp(-k_1 t)] \quad (S8)$$

$$q_t = \frac{k_2 . q_e^2 . t}{1 + q_e . k_2 . t} \quad (S9)$$

$$q_t = q_e . [1 - exp\,(-k_{AV} . t)^{nAV}] \quad (S10)$$

Where t is the contact time (min); q_t, and q_e are the amount of adsorbate adsorbed at time t and the equilibrium, respectively (mg g^{-1}); k_1 is the pseudo-first-order rate constant (min^{-1}); k_2 is the pseudo-second-order rate constant (g mg^{-1} min^{-1}); k_{AV} is the Avrami-fractional-order constant rate (min^{-1}), n_{AV} is the Avrami exponent (n >0).

Langmuir, Freundlich, and Liu's models were employed to analyze equilibrium data. Equations S11, S12 and S13 show the corresponding Langmuir, Freundlich, and Liu models (Lima, E.C. et al., 2021a).

$$q_e = \frac{Q_{max}.K_L.C_e}{1 + K_L.C_e} \quad \text{(S11)}$$

$$q_e = K_F.C_e^{1/nF} \quad \text{(S12)}$$

$$q_e = \frac{Q_{max}.(K_g.C_e)^{nL}}{1 + (K_g.C_e)^{nL}} \quad \text{(S13)}$$

Where q_e is the adsorbate amount adsorbed at equilibrium (mg g^{-1}); C_e is the adsorbate concentration at equilibrium (mg L^{-1}); Q_{max} is the maximum sorption capacity of the adsorbent (mg g^{-1}); K_L is the Langmuir equilibrium constant (L mg^{-1}); K_F is the Freundlich equilibrium constant [mg.g^{-1}.(mg.L^{-1})$^{-1/nF}$]; K_g is the Liu equilibrium constant (L mg^{-1}); n_F and n_L are the exponents of Freundlich and Liu model, respectively, (n_F and n_L are dimensionless).

2.6 Adsorption thermodynamics

Thermodynamic studies for the CIP adsorption onto R_1 and R_2 adsorbents were performed at temperatures ranging from 10ºC to 45°C (283 to 318K).

The Gibb's free energy change (ΔG^0, kJ mol^{-1}), enthalpy change (ΔH^0, kJ mol^{-1}), and entropy change (ΔS^0, J mol^{-1}K^{-1}) were evaluated with the aid of Equations S14–S17, respectively (Lima, E.C. et al., 2019a, 2019b).

$$\Delta G^0 = \Delta H^0 - T.\Delta S^0 \quad \text{(S14)}$$

$$\Delta G^0 = -RT.LnK_e^0 \quad \text{(S15)}$$

$$K_e^0 = \frac{(1000.K_g.Mw.[adsorbate]^0}{\gamma} \quad \text{(S16)}$$

The combination of Equations S14 and S15 leads to equation S17

$$LnK_e^0 = \frac{\Delta S^0}{R} - \frac{\Delta H^0}{R}.\frac{1}{T} \quad \text{(S17)}$$

R is the universal gas constant (8.314 J K^{-1} mol^{-1}); T is the absolute temperature (Kelvin); M_w is the molecular weight of the adsorbate (g mol^{-1}), $[adsorbate]^0$ is the standard molar concentration of the adsorbate, which by definition is 1 mol L^{-1}; γ is the activity coefficient of

the adsorbate. K_e^0 is the thermodynamic equilibrium constant, calculated according to equation S16. K_e^0 is dimensionless (Lima, E.C. et al., 2019a, 2019b).

K_e^0 is calculated by converting K_g values (Liu equilibrium constant) or K_L (Langmuir equilibrium constant), expressed in L mg^{-1} into L mol^{-1}. Firstly, the value K_g or K_L is multiplied by 1000 (mg g^{-1}), and then multiplied by the molecular weight of the adsorbate (g mol^{-1}) and by the standard concentration of the adsorbate (1 mol L^{-1}) and divided by the activity coefficient of the adsorbate (γ- dimensionless) (Lima, E.C. et al., 2019a, 2019b). It is assumed that the solution is sufficiently diluted to consider that the γ is unitary. Making these calculations, K_e^{Θ} becomes dimensionless (Lima, E.C. et al., 2019a, 2019b, 2020).

Equation S17 is the linearized van't Hoff equation (Lima, E.C. et al., 2020). On the other hand, recently, Lima et al. 2020 proposed using the nonlinear van't Hoff equation, as presented in equation S18.

$$K_e^0 = exp\left[\frac{\Delta S^0}{R} - \left(\frac{\Delta H^0}{R}\right).\frac{1}{T}\right] \qquad \text{(S18)}$$

Table S1. Chemical composition of simulated synthetic effluent.

**Phenolic compounds**	**Concentration (mg L^{-1})**
Catechol	100
2-nitrophenol	20
4-nitrophenol	20
4-Bromophenol	20
2,4-dinitrophenol	20
**Other organics**	
Sucrose	50
Glucose	50
Urea	20
Citric acid	20
Humic acid	20
Sodium Lauryl sulfate	10
**Inorganics**	
Ammonium phosphate	30
Sodium sulfate	20
Sodium carbonate	20
Sodium chloride	70
Sodium carbonate	20
Calcium nitrate	20
Potassium nitrate	20
Magnesium chloride	20
pH	7

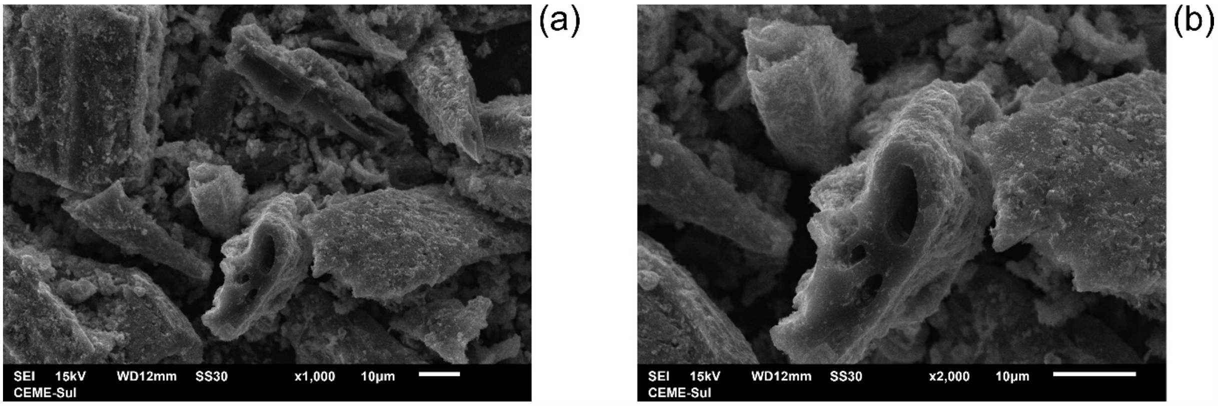

Figure S1. SEM images of ABC material.

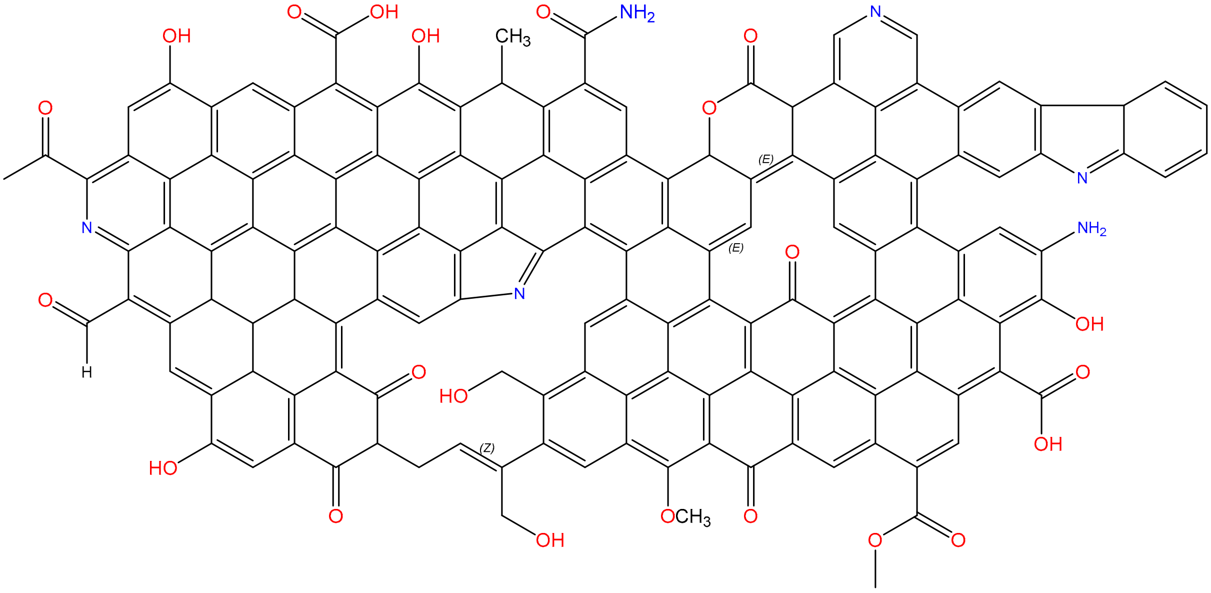

Figure S2 Proposed chemical structure for the ABC.
